# Environmental Attitudes in 28 European Countries Derived From Atheoretically Compiled Opinions and Self-Reports of Behavior

**DOI:** 10.3389/fpsyg.2022.875419

**Published:** 2022-07-05

**Authors:** Jan Urban, Florian G. Kaiser

**Affiliations:** ^1^Environment Centre, Charles University, Prague, Czechia; ^2^Global Change Research Institute, Czech Academy of Sciences, Brno, Czechia; ^3^Institute of Psychology, Otto-von-Guericke University Magdeburg, Magdeburg, Germany

**Keywords:** environmental attitude, attitude measurement, attitude-behavior consistency, Campbell paradigm, green consumption, cross-cultural comparison

## Abstract

People differ in their personal commitment to fighting climate change and protecting the environment. The question is, can we validly measure people’s commitment by what they say and what they claim they do in opinion polls? In our research, we demonstrate that opinions and reports of past behavior can be aggregated into comparable depictions of people’s personal commitment to fighting climate change and protecting the environment (i.e., their environmental attitudes). In contrast to the commonly used operational scaling approaches, we ground our measure of people’s environmental attitudes in a mathematically formalized psychological theory of the response process—the Campbell paradigm. This theory of the response process has already been extensively validated, and its relevance for manifest behavior has repeatedly been shown as well. In our secondary analysis of Eurobarometer data (*N* = 27,998) from 28 European countries, we apply the Campbell paradigm to a set of indicators that was not originally collected to be aggregated into a single scale. With our research, we propose a distinct way to measure behavior-relevant environmental attitudes that can be used even with a set of indicators that was originally atheoretically compiled. Overall, our study suggests that the Campbell paradigm provides a sound psychological measurement theory that can be applied to cross-cultural comparisons in the environmental protection domain.

## Introduction

Only a sound understanding of the extent to which people are motivated to endure inconveniences and other obstructions to fight climate change and to protect the environment will allow policy-makers to develop well-targeted policies that can promote sustainable lifestyles without provoking disruptive resistance in the populace. In our research and in contrast to the operational scaling approaches typically found in environmental protection research [see, e.g., Dunlap et al. (2000) and Milfont and Duckitt (2004)], we employ a *psychological* measurement theory called the Campbell paradigm [see, e.g., Kaiser et al. (2010) and Kaiser and Wilson (2019)]. The Campbell paradigm models the response process by linking the *measurand* (i.e., the latent attribute to be measured) with people’s manifest responses [i.e., verbally expressed opinions and self-reports of behavior; see Mari and Wilson (2014) and Mari et al. (2021); see also Chapter 6 in Borsboom (2005)]. Thus, our measure describes people’s *latent* commitment in terms of the probability that they will agree with *manifest* statements about environmental protection. As such, the measure is defined by the specific response process rather than being operationally defined by a fixed set of indicator items [see DeHouwer et al. (2013)]. But before we get to the theoretical details of the Campbell paradigm, we have to describe the conceptual issues involved in the measurement of personal commitment.

People differ in their personal *commitment* to and in their *opinions* about fighting climate change and protecting the environment. Commitment refers to the motivational force behind what people do to protect the environment [see, e.g., Kaiser et al. (2010, 2013)]—people’s behavioral propensity [see, e.g., Campbell (1963) and DeFleur and Westie (1963)]. By contrast, opinions refer to what people think about protecting the environment [for examples, see, e.g., Bogner and Wiseman (1999) and Dunlap et al. (2000)]. Specifically, opinions represent propositional thoughts that link an object (e.g., climate or environmental protection) with some attribute [e.g., necessary or urgent; see, e.g., Eagly and Chaiken (1993)]. These thoughts are typically expressed as self-referential statements of the form “I find protecting the environment to be important” [see, e.g., Lalljee et al. (1984) and Krosnick et al. (2005)].

Verbally stated opinions are frequently used to measure people’s environmental attitudes [see, e.g., Dunlap et al. (2000) and Milfont and Duckitt (2004)]. This practice persists despite the fact that opinions often do not correspond well with people’s behavior. For instance, in one study, the majority of participants expressed the opinion that it is everyone’s responsibility to pick up litter, but only a minority actually did so [see Bickman (1972)]. And even though the majority of participants in Diekmann’s (1996) Swiss sample stated the opinion that they generally act pro-environmentally, only a minority reported actually turning down the thermostat when they left their homes for more than 4 h. Verbally expressed opinions and reports of past behavior—even when they are a valid reflection of what people think and claim they do—are therefore not automatically valid measures of people’s commitment to protecting the environment (Kaiser et al., 2021), the motivational essence represented by people’s environmental attitudes [see, e.g., Kaiser et al. (2010, 2013)].

The question that we must ask is, are verbally expressed opinions and behavioral claims capable of capturing people’s personal commitment to fighting climate change and protecting the environment, that is, the commitment that ultimately surfaces in people’s manifest behavior? In other words, are opinion-poll-based measures of people’s environmental attitudes predictive of their manifest environmental protection? To date, the evidence has been mixed.

Whereas, environmental attitude—grounded in operational (rather than in psychological) measurement models in environmental protection research—has been found to at least account for intention measures and behavioral self-reports [see, e.g., Milfont and Duckitt (2004) and Bamberg and Möser (2007)], it has done so only inconsistently in cross-cultural research [see Marquart-Pyatt (2012); Morren and Grinstein (2016), and Tam and Chan (2017)]. In other words, rather surprisingly, people’s commitment as reflected by environmental attitude does not even always translate into behavioral self-reports [see, e.g., Eom et al. (2016) and Evans et al. (2018)]. Additionally, operational attitude measures (either as scales or as single-item measures) have often been found to fail to account for *specific manifest* environmentally protective behaviors in the field or lab [see, e.g., Bickman (1972) and Smith and Bell (1992); for a similar conclusion, see Kaiser and Byrka (2015)]. Not surprisingly, environmental attitude is distrusted by many regarding its relevance for manifest environmentally protective behavior [see, e.g., Stern (2000), Kollmuss and Agyeman (2002), and Gifford (2014)]. Part of the problem seems to be that opinion-based measures cannot reliably differentiate people with comparatively strong environmental attitudes [see Zhu and Lu (2017)].

By contrast, when environmental attitude is grounded in a psychological measurement theory that specifically models the response process by linking people’s latent environmental attitudes with their verbally expressed opinions and self-reports of behavior [i.e., the Campbell paradigm; see, e.g., Kaiser et al. (2010) and Kaiser (2021)], environmental attitude has repeatedly been shown to predict *manifest* environmentally protective behavior [see, e.g., Kaiser and Byrka (2015); Taube et al. (2018), Taube and Vetter (2019), and Kaiser et al. (2020)] and vice versa. Engagement in a manifest behavior has also been shown to predict people’s environmental attitudes [see Kaiser et al. (2021) and Kaiser and Lange (2021)]. Quite logically, people’s environmental attitudes, grounded in the Campbell paradigm, have also revealed a statistically significant negative association with the same people’s electricity consumption [see Arnold et al. (2018)]. Additionally, environmental attitude has been shown to be relevant for saving energy [see Henn et al. (2019)] and for seeking information about the science behind climate change [see Taube et al. (2021)]. Next, we move on to the specifics of the psychological measurement theory, the Campbell paradigm, and how it can be used to theoretically anticipate the response process.

According to Kaiser (2021), identifying people’s commitment to their personal environmental protection goal (people’s environmental attitudes, the *latent* psychological attribute) requires researchers to consider the persistence and the consistency of people’s goal striving. In other words, when people aim to protect the environment, they typically have to use a variety of behavioral means that are instrumental to pursuing that very goal. Not only must they ride a bike, but they must also recycle cardboard, avoid foods that are particularly environmentally harmful (e.g., meat), and refrain from owning a car (Kaiser et al., 2013). Consequently, to identify people’s commitment to a goal, researchers need to monitor arrays of behavior that are necessary for goal attainment. This is why measures of people’s environmental attitudes within the Campbell paradigm typically [but not exclusively, see Kaiser et al. (2018) and Kaiser and Wilson (2019)] consist of self-reports of past environmentally protective behaviors [i.e., the General Ecological Behavior scale; e.g., Kaiser and Wilson (2004) and Kaiser et al. (2007)].

According to the Campbell paradigm, people are presumed to be rational in an outcome-oriented or benefit-oriented sense. The underlying principle behind this notion of rationality is the optimization of an actor’s benefits and not the accuracy of a decision-maker’s processing of information [see Otto et al. (2014), Footnote 2]. Accordingly, when confronted with the various behavioral means that are available for realizing their personal environmental protection goal, people are expected to choose the ones that will enable them to realize this goal in a cost-effective manner [see Kaiser et al. (2010)]. Thus, people’s commitment to environmental protection becomes clear in the face of the behavioral costs that they are willing to endure in order to achieve their protection goal [see also Campbell (1963) and Kaiser and Wilson (2019)]. Because people accept costs to an extent that is consistent with their *latent* environmental attitudes, the strength of an individual’s environmental attitude can be equated with the occurrence likelihoods of the means (the *manifest* behaviors) required to protect the environment [see, e.g., Kaiser et al. (2010) and Kaiser (2021)].

Within the Campbell paradigm, people’s manifest behavior as well as their manifest responses in surveys (i.e., verbally expressed opinions and behavioral self-reports) are expected to be a function of two components: (a) people’s commitment to environmental protection (i.e., their environmental attitudes) and (b) the composite of the costs represented by a specific manifest behavior, a verbally expressed opinion, or a behavioral self-report. Formally, the Campbell paradigm is expressed with Equation 1.


(1) $$\text{ln}\,\left(\frac{p_{i\,j}}{1-p_{i\,j}}\right)=\theta_{i}-\delta_{j}$$

In this equation [i.e., the Rasch model; for more details, see, Rasch (1960/1980); for a more recent account, see, e.g., Wilson (2005)], the natural logarithm of the ratio of the probability (*p*_*ij*_) of person *i*’s engagement relative to the probability of their non-engagement (1-*p*_*ij*_) in a specific behavior *j* (i.e., its odds) is the result of the *difference* between *i*’s attitude (θ*_*i*_*) and the costs of behavior *j* (δ*_*j*_*). This is equivalent to saying that the strength of a person’s environmental attitude has to offset the specific costs of a particular protective behavior or of an attitude-relevant response in a survey before the behavior or the response has a reasonable chance (*p*_*ij*_ ≥ 0.50) of being implemented or endorsed by a person (Kaiser et al., 2010; Kaiser and Wilson, 2019). Even a comparatively undemanding response in a survey (e.g., expressing the opinion that environmental protection is important) is unlikely to become endorsed when a respondent is not at all committed to environmental protection.

In this research, we applied the Campbell paradigm to a set of indicators that were not originally collected to be aggregated into a single measurement instrument. In the process, we aggregated verbally expressed opinions and reports of past behavior into a valid measure of the strength of people’s environmental attitudes (representing people’s commitment to protecting the environment). On the basis of our psychological measurement theory—the Campbell paradigm (Kaiser et al., 2010; Kaiser and Wilson, 2019), we further expected to find people’s propensities for green consumption to *consistently* correspond with their environmental attitudes. Despite any intercultural and intracultural individual differences in people’s environmental attitudes, we anticipated a universal positive relationship between people’s environmental attitudes and their green consumption. With our analysis, we add to the still deficient amount of research that has compared the strength of the attitude-behavior relationship in different societies [see Milfont and Markowitz (2016)].

When researchers wish to compare measures cross-culturally, measurement instrument equivalence is a major concern [see Van de Vijver and Leung (2011)]. As such formal equivalence has proven difficult to attain in cross-cultural environmental psychology as well [see, e.g., Milfont et al. (2006) and Urban and Ščasný (2012)], we decided to employ *random item effects modeling* [for more details, see De Jong et al. (2007); see also Hartig et al. (2020)]. Random item effects modeling represents an alternative approach that is still suitable for attaining comparable environmental attitude measures without requiring formal equivalence [see, e.g., De Jong et al. (2007)]. Random item effects models are grounded in item response theory, and they are similar to structural equation models that approximate equivalence [see Cieciuch et al. (2018)]. To our knowledge, we are the first to use De Jong et al.’s (2007) modeling suggestion to compare environmental attitudes in cross-cultural research. Specifically, we calibrated a cross-cultural measure of environmental attitude using a mixed Rasch model.

In our secondary analysis of Eurobarometer data from 28 European countries, we developed an impromptu Campbellian measure of environmental attitude from an atheoretically compiled set of self-reports of environmentally protective behaviors and verbally expressed opinions concerning environmental protection. In doing so, we aggregated people’s opinions and self-reports of their past behavior into a measure of the strength of people’s commitment to protecting the environment (i.e., people’s environmental attitudes). Such a measure is—due to its formal characteristics as a Rasch scale—applicable in cross-cultural comparisons. Subsequently and analogous to previous studies that demonstrated the actual behavioral relevance of Campbell-paradigm-based attitude scales [see, e.g., Taube and Vetter (2019) and Kaiser et al. (2020)], our study—due to a lack of manifest behavioral criteria—at the very least demonstrates that people’s environmental attitudes reliably account for the same people’s propensity to engage in green consumption within and across countries.

## Materials and Methods

For our reanalysis, we used freely available data from the Eurobarometer 83.1 survey (European Commission, 2017). The data were collected in 2014 using a three-stage stratified random sampling approach. In the first stage, a number of sampling points were drawn with probabilities proportional to the population size and population density in each country. In the second stage, and beginning with an incidentally selected first address, specific addresses were determined using standard random route procedures [for details, see Kish (1995)]. In the third stage, participants were chosen from each household at random, using the closest birthday rule. All interviews were conducted face-to-face in people’s homes and in their national language.

### Participants

The Eurobarometer 83.1 covered the populations of the 28 member states of the European Union (EU) at the time when the data were collected (2014). Despite the sophisticated sampling procedure, we have reason to suspect that random sampling was not entirely successful as some of the national samples substantively departed from the population statistics in terms of gender, age, region, and size of locality. Consequently, we decided to reweight the national samples by gender, age, region, and size of locality so that they became more similar on these four variables to the respective national populations aged 15 and older. Note that reweighting did not significantly affect the substantive findings of our research.

The final dataset consisted of 27,998 participants. Of these, 14,692 (52.5%) were women. On average, the participants were 46.98 years old (*SD* = 18.43). The 28 national subsamples had between 500 (Cyprus) and 1,546 (Germany) participants (for more details about the national subsamples, see Supplementary Table 1).

### Measures

Without a practically or logically discernable behavioral criterion, we decided to develop two separate impromptu instruments: a Campbell-paradigm-based Rasch scale for people’s *environmental attitude* and a Guttman index for people’s *propensity to engage in green consumerism*. When attitude is measured through verbal behavior (i.e., opinions and self-reports of past behavior) as is done within the Campbell paradigm, the separation between behavioral indicator and behavioral criterion is arbitrary and can be attributed to research ambition [see Kaiser and Wilson (2019)]. In other words, we could just as well have used all available items to measure people’s environmental attitude. By doing so, however, we would have lost the opportunity to subsequently validate the newly developed environmental attitude measure.

For the measure of environmental attitude, we calibrated the 20 items shown in Supplementary Table 2, all related to environmental protection. Seven of these environmentally protective responses were self-reports of environmentally protective behavior (e.g., “Have you used your car less for environmental reasons in the past month?”), and 13 were opinions expressing support for environmental protection (e.g., “The EU should help non-EU countries improve their environmental standards”). Importantly, seven of these 20 survey items (Items 8, 14, 16–20 in Supplementary Table 2) closely corresponded with behavioral self-reports that are typically used with Campbell-paradigm-based environmental attitude measures [see, e.g., Kaiser and Wilson (2004)]. The majority of the remaining items expressed opinions typically employed in more traditional environmental attitude measures [see, e.g., Dunlap et al. (2000) and Milfont and Duckitt (2004)].

To reduce measurement error, all polytomous items were converted into a dichotomous format, with responses representing the environmentally protective option coded as 1 and responses representing the environmentally harmful option coded as 0 (see Supplementary Table 2 for more details about the specific coding used with each item). Note that reducing the absolute number of response options before calibrating a scale is a well-established and justified approach for preventing unreliable measurement due to excessive measurement error [for more details and some supporting evidence, see Kaiser and Lange (2021)]. Note that this procedure must not be confused with dichotomizing a continuous attitude scale after calibrating the measurement instrument (DeCoster et al., 2009). Note also that dichotomization is furthermore convenient from a technical point of view as it greatly simplifies the measurement model [see De Boeck and Wilson (2011)].

People’s green consumption propensity was measured with a Guttman index composed of two items: an intention item (i.e., “Are you willing to buy environmentally friendly products even if they cost a little bit more?” originally measured on a 4-point Likert scale and dichotomized by merging *totally disagree* with *tend to disagree* and *tend to agree* with *strongly agree*) and a behavioral self-report (i.e., “Have you bought environmentally friendly products marked with an environmental label during the past month for environmental reasons?”). With these rather simple indicators, we were able to differentiate between (a) people who did not intend to and did not purchase green products (i.e., low propensity for green consumption: between 5.2 and 37.6% of the national samples), (b) people who intended to but did not purchase green products (moderate propensity for green consumption: between 36.6 and 65.1% of the national samples), and (c) people who intended to purchase and actually purchased green products (high propensity for green consumption: between 8.0 and 57.8% of the national samples). Responses to the two items that would have violated a Guttman structure—that is, people who did not intend to purchase but actually purchased green products—were rare: between 0.2 and 2.4% of the national samples. This marginal proportion of Guttman errors speaks of the Guttman-structure consistency of the items [*C*_*R*_ > 0.98; see, e.g., Edwards (1983)].

### Statistical Analysis

We cross-culturally calibrated the proposed impromptu Campbellian environmental attitude measure with the random item effects Rasch model [see De Jong et al. (2007); Fox (2010), and De Boeck and Wilson (2011)]. The model belongs to the family of hierarchical item response theory models and is typically implemented within a Bayesian framework. The random item effects Rasch model is an extension of the classical Rasch model (see Equation 1). It posits that the natural logarithm of the ratio (i.e., the odds) of the probability (*p*_*ijk*_) of person *i* providing an environmentally protective response *j* in country *k* relative to the inverse probability (1-*p*_*ijk*_) is a function of the *difference* between *i*’s attitude (θ_*i*_) and the costs of providing a specific environmentally protective response *j* in country *k* (δ_*jk*_: see Equation 2):


(2) $$\text{ln}\,\left(\frac{p_{i\,j\,k}}{1-p_{i\,j\,k}}\right)=\theta_{i}-\delta_{j\,k}$$

The unique feature of the random item effects model is that it assumes that the costs of providing a specific environmentally protective response *j* vary across countries due to unobserved country-specific factors. Hence, the costs of providing a response *j* in country *k* are assumed to be drawn from a normal distribution of costs across countries; this distribution has an unknown overall or grand mean and an unknown variance. Both the grand mean and variance can be estimated for each survey response if the measurement model is calibrated jointly across countries. Because country-specific costs represented by a specific response are modeled as individual instances from a distribution of costs, the various costs in the different countries can be placed on a single dimension and, thus, numerically compared.

People’s environmental attitudes were estimated without imposing any constraints. However, to identify the model, we had to impose a conventional “sum-to-zero constraint” on the within-country costs [see Fox (2010)]. Thus and even though the costs of environmentally protective responses were allowed to vary across countries, the costs of providing the 20 responses within each country were constrained to result in an average cost of zero logits. Logits are the units with which the proposed scale reflects people’s environmental attitudes and the costs of providing environmentally protective responses. Logits represent the natural logarithm of the odds represented in Equations 1 and 2.

As is recommended in the Bayesian framework [see Stan Development Team (2018)], we imposed *weakly informative* priors as an expression of our initial lack of expectations regarding attitudes and costs. Whereas, weakly informative priors provide a convenient way to facilitate model convergence, they expectedly—given the size of our data sets—do not affect later results (Gelman et al., 2014). Specifically, our priors express the expectation that the costs of expressing opinions and self-reporting engagement in behavior in the past are normally distributed with a mean of zero and standard deviation of 10. This would imply, for instance, that rather excessive logit values of –20 and +20 would still be plausible.

Following previous successful applications of random item effects models that also employed a Bayesian framework [e.g., Janssen et al. (2000); De Jong et al. (2007), and Fox (2010)], we estimated our model with the Hamiltonian MCMC algorithm in Stan [see Stan Development Team (2018)]. Uncertainties about parameter estimates are expressed as Bayesian 90% credible intervals [see Gabry and Goodrich (2018)]. Although conceptually different from confidence intervals, credible intervals are used in a similar fashion as confidence intervals to quantify parameter uncertainty.

To assess model fit, we considered global and specific fit. Global model fit was assessed with the leave-one-out cross-validation procedure. The procedure captures how well a model predicts one observation given the remaining data. The percentage of responses that are not well predicted by the model (*Pareto k* > 0.5) and the improvement between alternative models in the expected log predictive density (*ELPD*) of a model relative to its standard error are jointly used to assess model fit and to compare the predictive validity of models, respectively [for details, see Vehtari et al. (2017)].

Because our samples are comparatively large, we assessed specific fit based on mean square (*MS*) values [see Wilson (2005)]. *MS* values reflect the relative discrepancy between the Rasch model’s predicted and the observed responses. *MS* values—weighted by the item variance—that fall between 0.75 and 1.30 are regarded as acceptable for instruments used in the scientific exploration of empirical relationships [see Wright et al. (1994)]. Note that an *MS* value of 0.75 corresponds to 25% less variation than the amount expected by the model, and an *MS* value of 1.30 indicates 30% more variation in the data. The latter speaks of a deficiency in the model.

## Results

Our results are organized into three sections. In the first section, we present details about the calibration of the impromptu Campbellian environmental attitude measures for the 28 European countries under observation. In the second section, we compare people’s environmental attitudes in the 28 European countries. In the third section, we finally explore the relationships between environmental attitude and people’s green consumption propensity across the 28 European countries.

### Measurement of Environmental Attitude Across Countries

Overall, the random item effects model fits the 519,616 responses provided by 27,998 participants rather well (*Pareto k* ≤ 0.5, leave-one-out global fit test) in 98.1% of the cases. Complementary evidence of an acceptable model fit was provided by the mean square (*MS*) statistics. On average, *MS* values were 1.11 (*SD* = 0.11). In other words, items had on average 11% more random variation than could be explained by the Rasch model. However, this excess random variation was fairly low, as there were only 10 (out of 560—given the 20 responses in 28 countries) estimates of the costs of providing an environmentally protective response that exceeded the *MS* threshold of 1.30.

**FIGURE 1:**
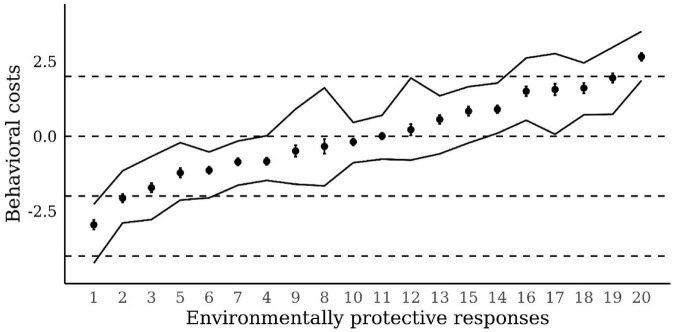
Costs of providing environmentally protective responses. Cost estimates of providing environmentally protective responses and their 90% credible intervals are denoted by points and error bars. The area between the two solid lines denotes the range of country-specific variability in the costs of the 20 responses that were considered. The response numbers correspond with the item numbers in Supplementary Table 2.

To test the equivalence and, thus, the comparability of the environmental attitude measures in the 28 European countries, we benchmarked a model that set the costs of providing the various environmentally protective responses equal across countries with a model that allowed the 20 costs to be specific to each of the 28 European countries. From the improved predictive validity of the model (Δ*ELPD* = 3,544, *SE* = 213), we were able to conclude that a model with country-specific costs fits the data considerably better than a model with the same cost estimates for all countries. Although the specific cost estimates varied considerably across countries (the area between the two solid lines in Figure 1), the trend of the cost estimates in the 28 countries nevertheless attest to the relatively stable ordering in the costs of the various environmentally protective responses across countries (see Figure 1).

From a technical point of view, the country-specific costs resulted in 28 nonequivalent and, thus, incomparable environmental attitude measures. Thus, the country-specific costs would traditionally have been regarded as bias, and comparing environmental attitudes across the 28 European countries would not have been possible. The situation was different here because we used the random item effects model [see De Jong et al. (2007)], as this particular model can accommodate country-specific costs.

### Environmental Attitude Across Countries

**FIGURE 2:**
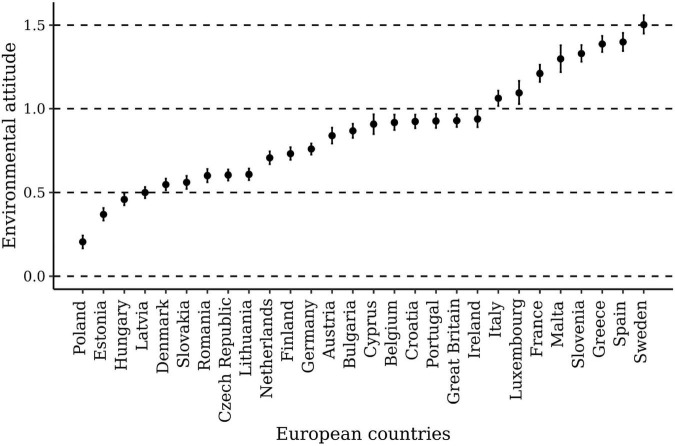
Environmental attitude in 28 European countries. The average attitudes are presented within their 90% credible intervals.

As suggested by the improvement in model fit, when attitudes were allowed to vary across countries, Δ*ELPD* = 13,219,171, *SE* = 15,566, people’s environmental attitudes differed significantly across countries. The precision of these country-typical attitude estimates (apparent in the 90% credible intervals) ranged from 0.07 to 0.16 logits, depending on the sizes of the national samples. The country-typical environmental attitudes in the 28 European countries ranged from 0.21 logits in Poland to 1.50 logits in Sweden (for more details, see Figure 2). Accordingly, the largest difference in people’s environmental attitudes between countries was 1.29 logits.

In all 28 countries, people’s environmental attitudes were unimodally distributed. They had a span of about 5 logits (see Supplementary Figure 1). The within-country variability was therefore about three times larger than the between-country variability. This means that a randomly chosen person living in the country with the lowest average attitude (i.e., Poland) still had a 40% chance of having a stronger environmental attitude than a randomly chosen person from the country with the highest average attitude (i.e., Sweden).

The fact that the average environmental attitude in the various countries was above zero (see Figure 2) suggests that the costs of providing environmentally protective responses were, on average, comparatively low in each national sample. This relative mismatch between attitudes and costs is somewhat undesirable because it could affect the attitude measure’s ability to differentiate between individuals with comparatively strong environmental attitudes. Nevertheless, we found country-specific reliabilities that were quite acceptable and spoke of the attitude measure’s ability to accurately differentiate among people with different environmental attitudes.

Separation reliabilities are computed as the ratio of two figures: (a) the observed variance of the attitude estimates minus the average squared standard error of these estimates and (b) the observed variance of the attitude estimates [see Wright and Masters (1982)]. Similar to the definition of reliability in classical test theory, the separation reliability captures the ratio of the true variance to the observed variance in the context of Rasch measurement. In our research, the reliabilities ranged from 0.67 in Cyprus to 0.78 in Austria (for the country-specific reliabilities, see Supplementary Table 3). Reliabilities between 0.70 and 0.80 are acceptable. Only two of the 28 reliabilities were marginally below the lower of the two thresholds.

### Environmental Attitude and Green Consumption Across Countries

**FIGURE 3:**
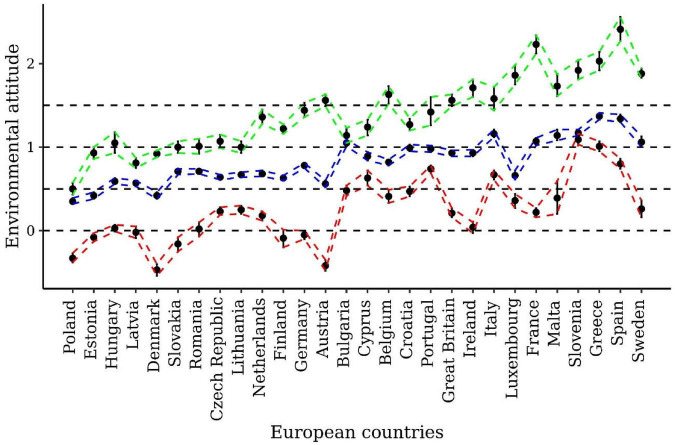
Average environmental attitudes of people with a 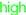, 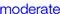, or 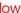 green consumption performance across 28 European countries. Countries are ordered according to their average environmental attitudes (see Figure 2). Dashed lines connect the 90% credible intervals of the three distinct green-consumption groups.

If our Campbellian measure of environmental attitude validly reflects people’s commitment to protecting the environment, we would expect to find people with a low (i.e., people who neither intended to purchase nor purchased green products), a moderate (i.e., people who intended to purchase but did not purchase green products), and a high propensity for green consumption (i.e., people who intended to purchase and actually purchased green products) to also systematically differ in their environmental attitudes [for evidence that our expectation holds, see Kaiser et al. (2021)]. In Figure 3, we can see that the average environmental attitudes in each of the three green consumption groups differed cross-culturally. Whereas, people who did not intend to buy and did not buy green products (i.e., people with a low propensity) typically had an environmental attitude below 0.80 logits across all countries, people who both intended to buy and bought green products (i.e., people with a high propensity) typically had an environmental attitude above 0.80 logits.

According to the posterior distribution of people’s environmental attitudes, we also found that people’s attitudes increased in the expected way (i.e., from low to high) with a very high probability (more than 99%) in the three green consumption groups in 26 out of 28 countries (see Figure 3). In the remaining two countries (i.e., Bulgaria and Slovenia), the probability of the average environmental attitude following the expected pattern in the three green consumption groups was still larger than 90% (compared with a chance probability of 17% that is given with six possible order permutations). Expectedly, we found support for the anticipated positive correlation between people’s environmental attitudes and their propensities for green consumption in all 28 European countries, *r*s = 0.19–0.43, all *p*s < 0.001 (for the country-specific correlations, see Supplementary Table 3).

## Discussion

In our secondary analysis of Eurobarometer data, we deliberately selected 20 opinions and behavioral self-reports to represent a Campbell-paradigm-based measure of environmental attitude (see Supplementary Table 2). From the sensible model fit, we conclude that the aggregation of the originally atheoretically compiled 20 responses into 28 reliable scales was successful. From these 28 confirmatory tests of the psychological measurement theory, we conclude that people’s response process seems to universally operate in the manner that is anticipated by the Campbell paradigm. The paradigm describes people’s affirmative opinion about environmental protection and their self-reports of past environmental protection as the result of two components: people’s commitment to the environmental protection goal (i.e., their environmental attitudes) and the costs reflected by a specific affirmation or a behavioral self-report [see, e.g., Kaiser and Wilson (2004), Kaiser et al. (2010, 2013), and Kaiser (2021)].

The 28 scales mirrored not only people’s opinions (i.e., what people said) but also what people claimed to do. By using these newly developed Campbell-paradigm-based measures, we were able to account for people’s self-declared green consumption in 28 European countries with small to medium effect sizes (see Supplementary Table 3). We found that the environmental attitudes of people with low, moderate, and high propensities for green consumption differed systematically in the countries we explored. These findings provide support for the theoretically expected association between people’s environmental attitudes (i.e., their commitment to protecting the environment) and their protective engagement [see also Kaiser and Byrka (2015); Byrka et al. (2017), Arnold et al. (2018); Taube et al. (2018), Taube and Vetter (2019), and Kaiser et al. (2020)]. More importantly and in contrast to other research [see, e.g., Marquart-Pyatt (2012); Eom et al. (2016), Morren and Grinstein (2016), and Tam and Chan (2017)], our findings corroborate a generalizable positive relationship between environmental attitude and engagement in environmentally protective behavior across a relatively large pool of countries. Our findings validate our newly developed scale as a measure of people’s commitment to fighting climate change and protecting the environment in the 28 European countries under exploration.

The mixed Rasch model that we used as our statistical model represents a general framework that allows for a single overall statistical test of the model. This is advantageous because a multitude of statistical tests—for each of the 28 countries—would have enhanced the risk of false-positive findings (Simmons et al., 2011). Even more importantly, the mixed Rasch model allowed us to confirm people’s universal response process anticipated by the Campbell paradigm separately in each country [for similar examples, see Gelman and Hill (2007)], and it allowed us to accommodate the substantial heterogeneity in the costs represented by the different verbally expressed opinions and behavioral self-reports across countries (which can be seen in the area between the two solid lines in Figure 1). Thus, the mixed Rasch model helped represent people’s environmental attitudes from various countries in a single metric and rendered the attitudes quantitatively comparable.

Across the 28 European countries, we found quite considerable variability of about 1.30 logits in people’s environmental attitudes with Poland and Sweden at the two extremes of the spectrum. When we contrasted this between-country variability with the within-country variability (i.e., at least five logits in each country; see Supplementary Figure 1), personal factors seemed to outperform societal factors by a multiplier of 3.5 in their ability to shape individual differences in people’s environmental attitudes. These results are in line with other studies that found that within-country (i.e., between-subjects) variability was several times larger than between-country variability [see, e.g., Gelissen (2007)]. In summary, both societal-level and individual-level factors fashion individual differences in people’s environmental attitudes but not in equal amounts.

Arguably, one of the advantages of the proposed response-aggregation approach that we presented is that it is grounded in a sound psychological theory of the response process. According to the Campbell paradigm, people’s responses are a function of the costs represented by a specific response and people’s attitudes (Kaiser et al., 2010; Kaiser and Wilson, 2019). By considering the costs of the responses, not only does this theoretical framework allow for the different types of responses that we typically find in opinion polls (e.g., evaluative statements, behavioral intentions, and behavioral self-reports), but even more importantly, it calls attention to actual costs in real-life contexts [see Kaiser and Lange (2021)].

As with all empirical research, our secondary analysis also comes with limitations. An important one is the impromptu nature of our measure of environmental attitude and, thus, its uncertain construct validity. One advantage of Rasch scales is that they are not bound to a particular set of indicator items to measure a certain attribute, for example, people’s environmental attitudes, as long as all indicator items can be modeled along a single dimension (Kaiser et al., 2018). As can be derived from the reasonable item fit statistics, this was the case for all items in all countries in our study. In other words, we have reason to believe that the 20 items fall along a single dimension in each of the 28 countries.

Seven of the indicator items we used (Items 8, 14, 16–20 in Supplementary Table 2) are behavioral self-reports, similar to those typically used with Campbell-paradigm-based measures of environmental attitude (Kaiser et al., 2013, 2018). The other 13 items concern explicit support for environmental protection (see Supplementary Table 2) and are similar to statements previously used with more traditional environmental attitude measures (Milfont and Duckitt, 2010). As such, the item content of our impromptu measure also appears to converge with other established environmental attitude measures. Finally, construct validity also comes with meaningful connections between our impromptu measure of environmental attitude and some external criteria from outside the measurement process itself (Whitely, 1977), such as when environmental attitude scores were found to be related to people’s propensity to engage in green consumption.

Another limitation concerns the fact that the national samples might not have been representative of their respective national populations in terms of environmental attitude. This concern arises because the national samples were already significantly different from their respective populations in terms of gender, age, region, and size of locality. Although we used reweighting procedures to alleviate this imbalance between samples and populations [e.g., Heeringa et al. (2010)], only random sampling procedures without additional post-stratification can eventually lead to more representative samples.

With our research, we were able to demonstrate that large-scale opinion polls in cross-cultural research can be reliably aggregated into valid depictions of people’s commitment to protecting the environment (i.e., the *motivational force* behind what people do to protect the environment). This is the case even though these polls were originally compiled atheoretically. In contrast to an *operational* scaling approach, we employed a *psychological* measurement theory that links people’s *latent* commitment to protecting the environment (i.e., their environmental attitudes) with people’s verbally expressed (*manifest*) opinions and self-reports of behavior [i.e., the Campbell paradigm; see, e.g., Kaiser et al. (2010) and Kaiser (2021)]. Such measures of environmental attitude have the extra benefit that they have repeatedly been shown to surface in people’s actual behavior [see, e.g., Taube et al. (2018); Taube and Vetter (2019), and Kaiser et al. (2020)]. The approach presented in this study is useful for the measurement and comparison of people’s commitment to protecting the environment, even with samples from various distinct cultural contexts. As such, it holds promise for more cross-cultural and cumulative environmental protection research.

## Data Availability Statement

Publicly available datasets were analyzed in this study. The data can be found here: https://osf.io/qehfj/?view_only=4fdd623f27914d51b9633ee148d8f538.

## Ethics Statement

Ethical review and approval was not required for the study on human participants in accordance with the local legislation and institutional requirements. Written informed consent from the patients/participants OR patients/participants legal guardian/next of kin was not required to participate in this study in accordance with the national legislation and the institutional requirements.

## Author Contributions

All authors listed have made a substantial, direct, and intellectual contribution to the work, and approved it for publication.

## Conflict of Interest

The authors declare that the research was conducted in the absence of any commercial or financial relationships that could be construed as a potential conflict of interest.

## Publisher’s Note

All claims expressed in this article are solely those of the authors and do not necessarily represent those of their affiliated organizations, or those of the publisher, the editors and the reviewers. Any product that may be evaluated in this article, or claim that may be made by its manufacturer, is not guaranteed or endorsed by the publisher.
