# Supplementary material for: Environmental Attitudes in 28 European Countries Derived From Atheoretically Compiled Opinions and Self-Reports of Behavior
Source: Front Psychol. 2022 Jul 5;13:875419. doi: 10.3389/fpsyg.2022.875419 (PMC9295715; doi:10.3389/fpsyg.2022.875419)
Supplement: Supplementary file 1 [file Data_Sheet_1.docx]

Supplementary Material

**Table S1.** Sociodemographic characteristics of the 28 national samples.

| Country | *M*_age_ (*SD*) | Women (%) | Tertiary education (%) | Sample size |
| --- | --- | --- | --- | --- |
| Austria | 46 (18) | 51 | 13 | 1,000 |
| Belgium | 48 (19) | 52 | 21 | 1,007 |
| Bulgaria | 48 (18) | 55 | 23 | 1,066 |
| Cyprus | 43 (18) | 47 | 27 | 500 |
| Czech Republic | 45 (17) | 52 | 11 | 1,056 |
| Germany | 49 (19) | 51 | 9 | 1,546 |
| Denmark | 48 (18) | 53 | 7 | 1,007 |
| Estonia | 47 (19) | 56 | 18 | 1,043 |
| Spain | 48 (18) | 54 | 14 | 1,039 |
| Finland | 48 (19) | 53 | 8 | 1,016 |
| France | 47 (20) | 51 | 8 | 1,027 |
| Great Britain | 46 (19) | 53 | 19 | 1,306 |
| Greece | 48 (19) | 52 | 30 | 1,008 |
| Croatia | 47 (18) | 54 | 14 | 1,012 |
| Hungary | 48 (19) | 53 | 14 | 1,085 |
| Ireland | 45 (17) | 50 | 21 | 1,005 |
| Italy | 48 (18) | 49 | 37 | 1,007 |
| Lithuania | 48 (19) | 55 | 17 | 1,013 |
| Luxemburg | 45 (18) | 49 | 14 | 504 |
| Latvia | 46 (17) | 57 | 22 | 1,002 |
| Malta | 46 (19) | 50 | 37 | 501 |
| Netherlands | 48 (19) | 53 | 8 | 1,032 |
| Poland | 45 (17) | 50 | 15 | 1,001 |
| Portugal | 47 (19) | 54 | 27 | 1,008 |
| Romania | 47 (18) | 55 | 32 | 1,062 |
| Sweden | 49 (18) | 53 | 16 | 1,025 |
| Slovenia | 48 (18) | 51 | 20 | 1,063 |
| Slovakia | 45 (17) | 53 | 25 | 1,057 |

**Table S2.** Opinions and self-reports of past behavior used to measure environmental attitude.

| Item number | | Item | Responses coded 1  (i.e., environmentally protective) | |  |
| --- | --- | --- | --- | --- | --- |
| 1 | How important is protecting the environment to you personally? [4-point Likert-type response scale] | | | Very important; fairly important | |
| 2 | More EU funding should be allocated to supporting environmentally friendly activities. [4-point Likert-type response scale] | | | Totally agree; tend to agree | |
| 3 | The efficient use of natural resources can boost economic growth in the EU. [4-point Likert-type response scale] | | | Totally agree; tend to agree | |
| 4 | In your opinion, to what extent do the following factors influence your quality of life? State of the environment. [4-point Likert-type response scale] | | | Very much; quite a lot | |
| 5 | The protection of the environment can boost economic growth in the EU. [4-point Likert-type response scale] | | | Totally agree; tend to agree | |
| 6 | In your opinion, is each of the following currently doing too much, doing about the right amount, or not doing enough to protect the environment? Big companies and industry. | | | Not doing enough | |
| 7 | The EU should help non-EU countries improve their environmental standards. [4-point Likert-type response scale] | | | Totally agree; tend to agree | |
| 8 | Have you separated most of your waste for recycling for environmental reasons in the past month? [yes-no] | | | Yes | |
| 9 | In your opinion, is each of the following currently doing too much, doing about the right amount, or not doing enough to protect the environment? The national government. | | | Not doing enough | |
| 10 | In your opinion, is each of the following currently doing too much, doing about the right amount, or not doing enough to protect the environment? Citizens themselves. | | | Not doing enough | |
| 11 | Thinking about how the public authorities in your country spend money and what they invest in, which of the following two statements comes closest to your view? [1 choice out of 2 statements] | | | Environmentally friendly considerations over cost considerations* | |
| 12 | In your opinion, is each of the following currently doing too much, doing about the right amount, or not doing enough to protect the environment? The European Union. | | | Not doing enough | |
| 13 | In your opinion, is each of the following currently doing too much, doing about the right amount, or not doing enough to protect the environment? Your region. | | | Not doing enough | |

continued

| Item number | | Item | Responses coded 1  (i.e., environmentally protective) | |  |
| --- | --- | --- | --- | --- | --- |
| 14 | Have you cut down on your energy consumption (e.g., by adjusting your air conditioning or heating, not leaving appliances on stand-by, buying energy-efficient appliances) for environmental reasons in the past month? [yes-no] | | | Yes | |
| 15 | In your opinion, is each of the following currently doing too much, doing about the right amount, or not doing enough to protect the environment? Your city, town, or village. | | | Not doing enough | |
| 16 | Have you chosen an environmentally friendly way to travel (by foot, bicycle, public transport) for environmental reasons in the past month? [yes-no] | | | Yes | |
| 17 | Have you chosen local products for environmental reasons in the past month? [yes-no] | | | Yes | |
| 18 | Have you cut down on your water consumption for environmental reasons in the past month? [yes-no] | | | Yes | |
| 19 | Have you reduced waste (e.g., by avoiding overpackaged products and buying products with a longer life) for environmental reasons in the past month? [yes-no] | | | Yes | |
| 20 | Have you used your car less for environmental reasons in the past month? [yes-no] | | | Yes | |

*Note.* ***** The other available response option for Item 12 was “Cost considerations over environmentally-friendly considerations.”

**Table S3.** Reliabilities of the environmental attitude measures and Pearson correlations (*r*) between environmental attitude and green consumption and their probabilities (*p*) in 28 European countries.

| Country | Reliability | *r* | *p* ≤ |
| --- | --- | --- | --- |
| Austria | .78 | .43 | .001 |
| Belgium | .76 | .27 | .001 |
| Bulgaria | .72 | .23 | .001 |
| Cyprus | .67 | .19 | .001 |
| Czech Republic | .74 | .26 | .001 |
| Germany | .77 | .34 | .001 |
| Denmark | .74 | .39 | .001 |
| Estonia | .73 | .32 | .001 |
| Spain | .74 | .31 | .001 |
| Finland | .76 | .33 | .001 |
| France | .76 | .41 | .001 |
| Great Britain | .77 | .31 | .001 |
| Greece | .75 | .25 | .001 |
| Croatia | .73 | .23 | .001 |
| Hungary | .76 | .29 | .001 |
| Ireland | .77 | .36 | .001 |
| Italy | .75 | .20 | .001 |
| Lithuania | .72 | .24 | .001 |
| Luxemburg | .75 | .37 | .001 |
| Latvia | .69 | .25 | .001 |
| Malta | .75 | .20 | .001 |
| Netherlands | .75 | .32 | .001 |
| Poland | .75 | .23 | .001 |
| Portugal | .72 | .15 | .001 |
| Romania | .74 | .23 | .001 |
| Sweden | .75 | .30 | .001 |
| Slovenia | .72 | .20 | .001 |
| Slovakia | .76 | .29 | .001 |

*Note.* Separation reliabilities were computed as the ratio of two figures: (a) the observed variance of the attitude estimates minus the average squared standard error of these estimates and (b) the observed variance of the attitude estimates (Wright & Masters, 1982). The interpretation of these reliabilities is similar to the interpretation of Cronbach’s alpha. Reliabilities between .70 and .80 are quite acceptable.

**Figure S1**

*Distributions of attitude levels in each of the 28 European Countries*

*
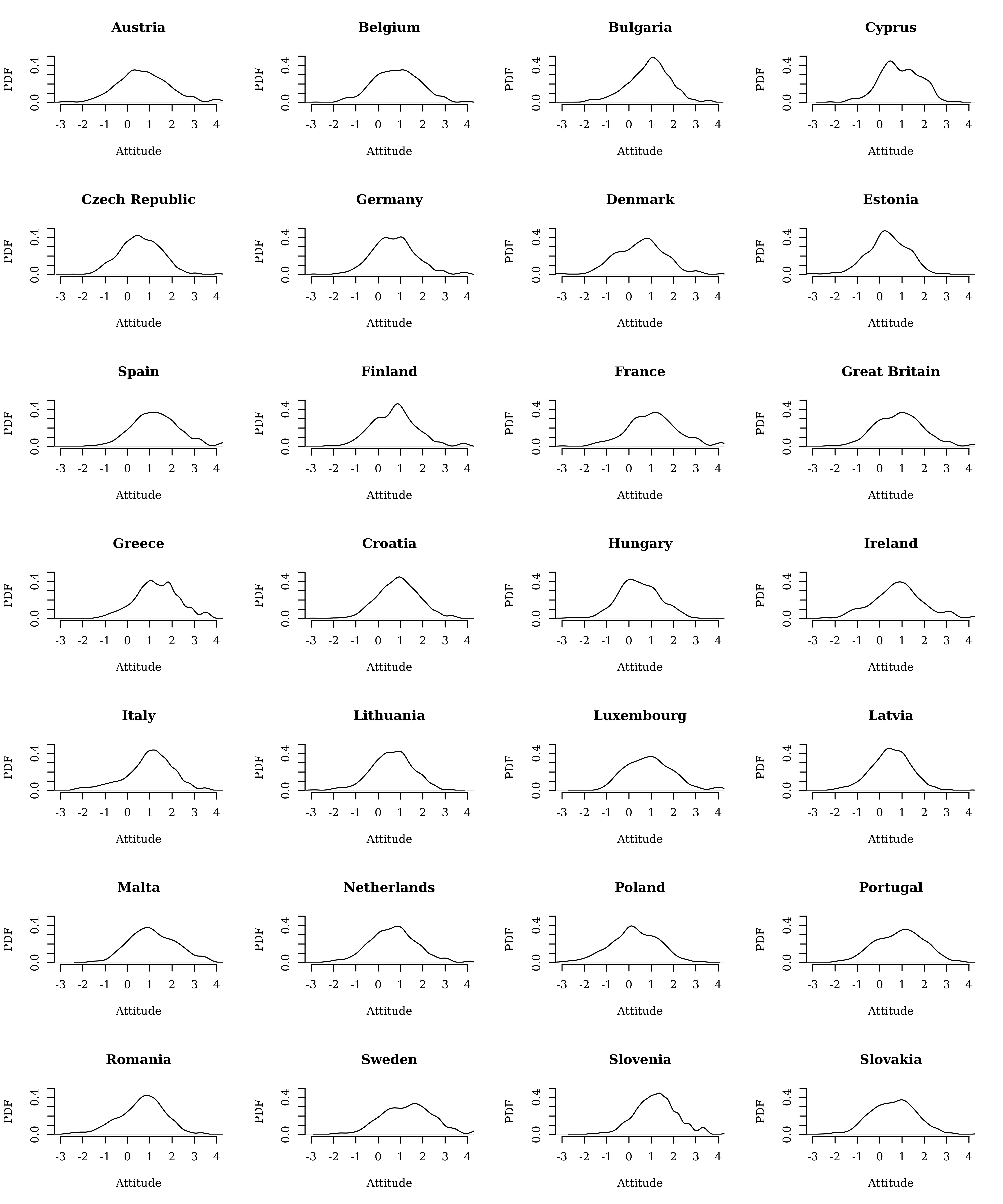
*

*Note.* Attitude levels are shown on the x-axis, probability density function (PDF) on the y-axis.
